# E-Mental Health Care Among Young Adults and Help-Seeking Behaviors: A Transversal Study in a Community Sample

**DOI:** 10.2196/jmir.4254

**Published:** 2015-05-15

**Authors:** Nadia Younes, Aude Chollet, Estelle Menard, Maria Melchior

**Affiliations:** ^1^Centre Hospitalier de VersaillesLe ChesnayFrance; ^2^EA 4047Université de Versailles Saint QuentinVersaillesFrance; ^3^INSERM, UMR_S 1136Institut Pierre Louis d’Épidémiologie et de Santé PubliqueÉquipe de Recherche en Epidémiologie SocialeF-75013, ParisFrance; ^4^Sorbonne Universités, UPMC Univ Paris 06UMR_S 1136, Institut Pierre Louis d’Épidémiologie et de Santé Publique, Équipe de Recherche en Epidémiologie SocialeF-75013, ParisFrance

**Keywords:** Internet, mental health services, young adult, epidemiology, health care disparities

## Abstract

**Background:**

The Internet is widely used by young people and could serve to improve insufficient access to mental health care. Previous information on this topic comes from selected samples (students or self-selected individuals) and is incomplete.

**Objective:**

In a community sample of young adults, we aimed to describe frequency of e-mental health care study-associated factors and to determine if e-mental health care was associated with the use of conventional services for mental health care.

**Methods:**

Using data from the 2011 wave of the TEMPO cohort study of French young adults (N=1214, aged 18-37 years), we examined e-mental health care and associated factors following Andersen’s behavioral model: predisposing factors (age, sex, educational attainment, professional activity, living with a partner, children, childhood negative events, chronic somatic disease, parental history of depression), enabling factors (social support, financial difficulties, parents’ income), and needs-related factors (lifetime major depression or anxiety disorders, suicidal ideation, ADHD, cannabis use). We compared traditional service use (seeking help from a general practitioner, a psychiatrist, a psychologist; antidepressant or anxiolytics/hypnotics use) between participants who used e-mental health care versus those who did not.

**Results:**

Overall, 8.65% (105/1214) of participants reported seeking e-mental health care in case of psychological difficulties in the preceding 12 months and 15.7% (104/664) reported psychological difficulties. Controlling for all covariates, the likelihood of e-mental health care was positively associated with 2 needs-related factors, lifetime major depression or anxiety disorder (OR 2.36, 95% CI 1.36-4.09) and lifetime suicidal ideation (OR 1.91, 95% CI 1.40-2.60), and negatively associated with a predisposing factor: childhood life events (OR 0.60, 95% CI 0.38-0.93). E-mental health care did not hinder traditional care, but was associated with face-to-face psychotherapy (66.2%, 51/77 vs 52.4%, 186/355, *P*=.03).

**Conclusions:**

E-mental health care represents an important form of help-seeking behavior for young adults. Professionals and policy makers should take note of this and aim to improve the quality of online information on mental health care and to use this fact in clinical care.

## Introduction

### Background

As written by Hartzband and Groopman [1]: “Medicine has built on a long history of innovation, from the stethoscope and roentgenogram to magnetic resonance imaging and robotics. Doctors have embraced each new technology to advance patient care. But nothing has changed clinical practice more fundamentally than one recent innovation: the Internet. Its profound effects derive from the fact that while previous technologies were fully under doctors’ control, the Internet is equally in the hands of patients. Such access is redefining the roles of physician and patient.” The main impact of the Internet is through the virtually exponentially growing amounts of heath information [2]. The proportion of households with Internet access in Europe is high: 73% are equipped, 68% of Europeans use the Internet at least once a week, and 56% use it daily, although significant disparities are reported between countries [3].

E-mental health care, defined as “mental health services and information delivered or enhanced through the Internet and related technologies,” comprises 4 areas: information provision, screening, assessment and monitoring, and intervention and social support [4]. At present, the Internet is ranked higher as a source of information than as a source to trust, with health professionals remaining the most trusted sources of information for mental health problems [5]. A population survey conducted in the United Kingdom reported that 10.6% of youths had used the Internet to find out about mental health and this level reached 20.5% among those with a history of mental health problems [6]. Potential benefits of online health-information seeking are easy accessibility; absence of geographical boundaries; free access; interactivity; potential social support, personalization, anonymity, and privacy; and potential to address the gap between identified needs and limited resources. Nevertheless, several concerns exist: disparities in access to computers and the Internet, quality of online health information even though several studies show that it is reasonable [7-9], financial interests, and competition with conventional services [2,4]. Furthermore, there is insufficient evidence that e-mental health interventions are efficacious and valid, especially social media and communication sites [10]. However, for psychiatric patients, the importance of the Internet is now unquestionable, regardless of diagnosis, primarily for information provision [11].

The Internet is seen as a major medium for reaching contemporary young people [12]. There is no consensus regarding the definition of young adulthood in the 2 dominant life models of adulthood defined by Erikson (age range 20-40 years) [13] and Levinson (20-24, 24-29, and 30-39 years) [14] and in research (eg, 18-25 [15], 16-20 [16], 18-34 [17], or 15-39 [18] years have previously been considered as young adults). A recent paper defined young adulthood as the period between ages 20 and 24 years, arguing about the relevance of this period regarding development: physical development (young women are typically fully developed physically; young men continue to gain height, weight, muscle mass, and body hair), cognitive development (ability to think ideas through from beginning to end, to delay gratification, to examine inner experiences, increased concern for the future, continued interest in moral reasoning), and social and emotional development (sense of identity, including sexual; increased emotional stability, concern for others, and independence and self-reliance; importance on peer relationships; more serious relationships; regain some interest in social and cultural traditions) [19].

Mental heath issues are challenging at that age because anxiety, mood, or substance use disorders tend to be frequent (75% of lifetime cases emerge by age 24 years, most anxiety disorders occur between 11 and 15 years, most substance-misuse disorders between 19 and 21 years, and mood disorders between 24 and 30 years [19-21]). Because of unsatisfactory mental health care access, only 18% to 34% of young people with high levels of symptoms of depression or anxiety seek professional help [15]. In the European Study of the Epidemiology of Mental Disorders (ESEMeD) survey conducted in Europe, compared to participants older than 65 years, participants aged 18 to 24 years were least likely to get care for mental health problems followed by participants aged 25 to 34 years [22]. Young adults born after 1993 (when the Internet became widely available) have been referred to as digital natives because they are fundamentally different from previous generations in that they grew up with the Internet. The Internet is completely natural to digital natives, is a key part of their lives, and a predominant source of health information [23]. A recent systematic review investigated the effectiveness of online services in facilitating mental health help seeking in young people aged 14-25 years [24]. In all, 38.4% reported seeking mental health information on the Internet, but these data came from selected samples (students or self-selected individuals) and from selected countries (9 studies were conducted within Australia; 3 in the United States; 2 in Canada; 1 in Germany, Ireland, Norway, United Kingdom; and none in France). This review concluded that online mental health services may conceivably assist in all elements of the help-seeking process and invited further research to examine the effectiveness of e-mental health care, how it interacts with face-to-face services, and whether the use of online services can overcome barriers to mental health care and increase help-seeking behavior.

Since this review was published, 2 studies provided more information about the use of the Internet by young adults for mental health difficulties. According to a Spanish study conducted among a sample of students, entering keywords into a search engine, portal, or Internet Service Provider was the most frequently used procedure, with usually no attention to the date or the origin of the information and with a strong distrust in online mental health information [25]. According to a Canadian study among youth aged 17-24 years (87% students), when using the Internet for information-seeking purposes, the 3 most common features were looking for information about symptoms (52.4%), for treatment options (47.4%), and for assessment tests (23.8%) [26]. In France, 99% of people aged 12-24 years and 96% of those aged 25-39 years have access to the Internet [27]. A nationally representative study conducted in France established that 48.5% of young adults aged 15-30 years used the Internet to look for health information and one-third report having changed their health behaviors because of their online searches; however, this study did not investigate e-mental health care [28].

### Objectives

In this investigation, conducted in a community sample of young adults in France, we aimed to (1) describe the frequency of e-mental health care for psychological difficulties; (2) determine associated health access factors according to Andersen’s behavioral model: predisposing, enabling, and needs-related factors [29]; and (3) determine the association between e-mental health care and conventional services.

## Methods

### Study Sample

Our sample comes from the Trajectoires Épidémiologiques en Population (TEMPO) study and comprised young adults defined as ages 18-37 years within the classes of the 2 dominant life models of adulthood: Erikson (age range 20-40 years) [13] and Levinson (20-24, 24-29, and 30-39 years) [14], and larger than a more recent definition (20-24 years) [19]. The TEMPO study launched in 2009 and includes a sample of young adults in France whose parents took part in the Gaz et Electricité (GAZEL) epidemiological cohort study, composed of 20,625 employees of a large French public sector utility company, Électricité de France-Gaz de France (EDF-GDF), the French national gas and electricity companies [30-37]. The TEMPO cohort has been described in detail elsewhere [38]. Briefly, the study was set up in 2009 among young adults aged 22-35 years, whose parents participated in the GAZEL cohort study and who took part in a study of children’s mental health in 1991 and 1999 (the GAZEL Youth study). In 2011, all TEMPO study participants and other offspring of GAZEL cohort participants aged 18-37 years were invited to take part in the TEMPO study. The 2011 sample (n=1214) included 526 individuals who took part in the 2009 TEMPO study assessment and agreed to be followed up (70.1% participation) and 688 new members (14.4% participation). In 2011, data were collected via a 30-minute phone interview assessing their health, health behaviors, access to health care, and socioeconomic and life circumstances. Study participants unable to take part in the phone interview were invited to complete the study questionnaire online [38-42]. The TEMPO study received approval from France’s national committees for data protection (Comité Consultatif sur le Traitement des Informations pour la Recherche en Santé, Commission Nationale Informatique et Liberté).

### Measures

E-mental health care was assessed by the following question: “In the preceding 12 months, did you consult the Internet for a psychological problem?” (yes/no/I did not have psychological problem). In addition, several factors potentially associated with e-mental health care use were investigated:

1. Predisposing factors: sex, age in 2011(<30 or ≥30 years; we chose arbitrarily to split at 30 years because it was in the middle of the age range of our sample), educational attainment (<high school diploma vs ≥high school diploma), family situation living with a partner (yes vs no), lifetime unemployment (yes vs no), childhood negative events (family conflicts, bullying, lack of affection; yes vs no), chronic somatic disease (obesity, diabetes, digestive disease, cancer, and epilepsy), and parental depression based on parents’ reports of treated depression on the yearly GAZEL study assessments from 1989 to 2011 (yes vs no) [43,44] and TEMPO participants’ reports regarding their parents on the Family Interview for Genetic Studies (yes vs no) [45].
2. Enabling factors: self-reported financial difficulties (yes vs no), self-reported income (<€2600 vs >€2600 per month), and social support as measured using the Berkman Social Networks and Social Support questionnaire (insufficient vs sufficient) [46].
3. Needs-related factors: presence of a mental health disorder ascertained using the Mini-International Neuropsychiatric Interview (MINI), a short structured clinical interview allowing researchers to diagnose psychiatric disorders according to the International Classification of Diseases (ICD-10). We assessed whether participants had major depression, panic, phobia, or generalized anxiety disorder in the preceding 12 months [47]. Additionally, lifetime suicidal ideation, attention deficit and hyperactivity disorder (ADHD) using the French version of the ADHD Self-Report Scale (ASRS) [48], and lifetime cannabis abuse were ascertained.

Help seeking from a general practitioner (GP), a psychiatrist, or a psychologist and antidepressant or anxiolytics/hypnotics use in lifetime were also assessed.

### Statistical Analysis

Following descriptive analyses, we tested associations between factors described in Andersen’s model and e-mental health care using (1) bivariate chi-square tests (categorical variables) and *t* tests (continuous variables) and (2) logistic regression models controlled for all factors associated with the study outcome in bivariate models (significant at *P*<.20 in order to control for as many potentially relevant covariates as possible). We repeated analyses weighing factors associated with study participation to verify the robustness of our results after correction for selection bias [49]. Then we compared traditional service use between participants who used online information versus those who did not with bivariate chi-square tests. Data were analyzed using SAS v9.3 (SAS Institute Inc, Cary, NC, USA).

## Results

Of the 1214 TEMPO Participants, 664 reported psychological difficulties and documented whether they used the Internet or not. Their mean age was 30.8 (SD 3.8) years, 72.7% (483/664) were female, 5.0% (33/657) were students, 63.7% (389/611) worked in a high occupational grade, 43.7% (290/664) lived with a partner, 66.0% (438/664) reported sufficient social support, and 12.4% (82/664) had a common mental health disorder (Table 1).

**Table 1:** Descriptive analyses of TEMPO young adults who reported psychological difficulties and comparison analyses between those who used and did not use e-mental health.

Associated factors			Total sample, n (%) N=664	E-mental health, n (%) n=104	No e-mental health, n (%) n=560	χ^2^ (*df*)^a^	*P*
**Predisposing factors**							
	**Age (years)**					0.5 (1)	.49
		<30	198 (29.8)	34 (17.2)	164 (82.8)		
		≥30	466 (70.2)	70 (15.0)	396 (85.0)		
	**Gender**					0.2 (1)	.69
		Female	483 (72.7)	74 (15.3)	409 (84.7)		
		Male	181 (27.3)	30 (16.6)	151 (83.4)		
	**Education**					0.5 (1)	.47
		High school or less	238 (35.8)	34 (14.3)	204 (85.7)		
		Beyond high school	426 (64.2)	70 (16.4)	356 (83.6)		
	**Marital status**					0.9 (1)	.34
		Living in couple	290 (43.7)	41 (14.1)	249 (85.9)		
		Living alone	374 (56.3)	63 (16.8)	311 (83.2)		
	**Students**					0.3 (1)	.56
		Yes	33 (5.0)	4 (12.1)	29 (87.9)		
		No	624 (95.0)	99 (15.9)	525 (84.1)		
	**Unemployed**					0.7 (1)	.40
		Yes	332 (50.1)	56 (16.9)	276 (83.1)		
		No	331 (49.9)	48 (14.5)	283 (85.5)		
	**Child event**					1.8 (1)	.18
		Yes	334 (50.3)	46 (13.8)	288 86.2		
		No	330 (49.7)	58 (17.6)	272 82.4		
	**Somatic chronic disease**					2.3 (1)	.13
		Yes	159 (24.0)	31 (19.5)	128 (80.5)		
		No	505 (76.1)	73 (14.5)	432 (85.5)		
	**Parental depression**					0.8 (1)	.38
		Yes	112 (20.0)	20 (17.9)	92 (82.1)		
		No	447 (80.0)	65 (14.5)	382 (85.5)		
**Enabling factors**							
	**Financial difficulties**					0.1 (1)	.82
		Yes	155 (23.4)	25 (16.1)	130 (83.9)		
		No	508 (76.6)	78 (15.4)	430 (84.7)		
	**Parental income (€)**					0.6 (1)	.43
		<2600	384 (59.6)	52 (13.5)	332 (86.5)		
		≥2600	260 (40.4)	41 (15.8)	219 (84.2)		
	**Social support**					0.7 (1)	.42
		Sufficient	438 (66.0)	65 (14.8)	373 (85.2)		
		Insufficient	226 (34.0)	39 (17.3)	187 (82.7)		
**Needs factors**							
	**Depression or anxiety disorder**					13.1 (1)	<.001
		Yes	82 (12.4)	24 (29.3)	58 (70.7)		
		No	582 (87.7)	80 (13.8)	502 (86.3)		
	**Lifetime cannabis use**					0.0 (1)	.99
		Yes	418 (63.3)	64 (15.3)	354 (84.7)		
		No	242 (36.7)	37 (15.3)	205 (84.7)		
	**ADHD**					3.3 (1)	.07
		Yes	57 (11.6)	14 (24.6)	43 (75.4)		
		No	435 (88.4)	66 (15.2)	369 (84.8)		
	**Suicidal ideation**					12.7 (1)	<.001
		Yes	260 (39.2)	57 (21.9)	203 (78.1)		
		No	404 (60.8)	47 (11.6)	357 (88.4)		

^a^ Chi-square test between young adults who reported using e-mental health care and young adults who did not use e-mental health care.

In all, 8.65% (105/1214) of study participants reported ever using the Internet for psychological difficulties. Among participants who reported psychological difficulties, the prevalence was 15.7% (104/664).

In the bivariate analyses (Table 1), e-mental health care was not significantly associated with any of the predisposing or enabling factors studied but was associated with 2 needs-related factors: the presence of a common depressive or anxious mental health disorder (*P*<.001) and lifetime suicidal ideation (*P*<.001).

In the multivariate analyses (Table 2), 3 variables were significantly associated with e-mental health care: childhood negative events (OR 0.6, 95% CI 0.38-0.93), the presence of a common mental health problems (OR 2.36, 95% CI 1.36-4.09), and lifetime suicidal ideation (OR 2.07, 95% CI 1.33-3.23). This model explained 9.3% of the total variance.

**Table 2:** Factors associated with seeking e-mental health care through multiple logistic regression analysis (N=664).

Factors		OR (95% CI)	*P*
**Gender**			.81
	Male	1	
	Female	0.95 (0.59-1.52)	
**Age (years)**			.52
	<30	1	
	≥30	0.86 (0.54-1.36)	
**Negative childhood event**			.02
	No	1	
	Yes	0.60 (0.38-0.93)	
**Anxiety or depressive disorder**			.002
	No	1	
	Yes	2.36 (1.36-4.09)	
**Suicidal ideation**			.001
	No	1	
	Yes	2.07 (1.33-3.23)	

Young adults who reported using e-mental health care were not different from those who did not report use in terms of actual help seeking from GPs or from psychiatrists, and in antidepressant or anxiolytics/hypnotics use. Young adults who reported using e-mental health care sought more help from psychologists than those without e-mental health care (66.2%, 51/77 vs 52.4%, 186/355, *P*=.03) (Table 3).

**Table 3:** E-mental health care use and seeking help in traditional health care (N=664).

Mental health care		E-mental health, n (%) n=104	No e-mental health, n (%) n=560	*P*
**GP help seeking**				.07
	No	25 (32.0)	154 (43.1)	
	Yes	53 (68.0)	203 (56.9)	
**Psychiatrist help seeking**				.43
	No	44 (59.5)	229 (64.3)	
	Yes	30 (40.5)	127 (35.7)	
**Psychologist help seeking**				.03
	No	26 (33.8)	169 (47.6)	
	Yes	51 (66.2)	186 (52.4)	
**Antidepressant use**				.99
	No	76 (73.1)	409 (73.0)	
	Yes	28 (26.9)	151 (27.0)	
**Anxiolytics/hypnotics use**				.11
	No	52 (50.0)	327 (58.4)	
	Yes	52 (50.0)	233 (41.6)	

## Discussion

### Principal Findings

In a community sample of French young adults, we found that 8.65% (105/1214) reported seeking e-mental health care in the preceding 12 months and this prevalence reached 15.7% (104/664) in the case of common mental health problems. The likelihood of e-mental health care was associated with 2 needs-related factors, common mental health problems (OR 2.36, 95% CI 1.36-4.09) and lifetime suicidal ideation (OR 2.07, 95% CI 1.33-3.23), and was negatively associated with a predisposing factor, childhood negative events (OR 0.6, 95% CI 0.38-0.93). E-mental health care does not hinder conventional care; young adults who reported using e-mental health care were not different from those who did not in terms of seeking help from a GP, a psychiatrist, or in terms of antidepressant or anxiolytics/hypnotics use. To the contrary, they sought help from psychologists more frequently than young adults who did not seek e-mental health care (66.2%, 51/77 vs 52.4%, 186/355, *P*=.03).

### Frequency of E-Mental Health Care

The frequency of e-mental health care use is lower than reported in other countries for young people (mean 38.4%, range 18%-53% according to a recent review) [24]. This may be due to differences between study samples (students, self-selected samples, sample drawn from the community with only 5% students and differences in age range; mean age of participants for the studies was 16.5-26.2 years vs 18-37 years in this study). Our percentage is closer to the 10.6% (20.0% in case of mental health problems) reported in England in young adults older than 18 years [6]. At the international level, lower Internet use has already been reported in France for health information provision among young people in a representative sample from the general population [28].

### Andersen’s Model for Young Adults’ E-Mental Health Care

Results about factors associated with Internet use are in-line with previous studies. We verified that needs factors are the most important, common mental health disorders and lifetime suicidal ideation, and contrary to previous studies, we measured for the first time mental health disorders with specific standardized tools [6,50]. Our results are also in-line with previous studies of Andersen’s model applied to traditional health care service utilization for the most prevalent mental disorders, showing 2 major needs-related factors (emotional problems, number of mental disorders) [29,51,52]. If personal income was a barrier in service utilization, it is not a barrier for Internet use [51]. Additionally, we did not find the gender barrier to online services reported elsewhere [12,24,25,28,50,53].

It is the first time that a study examined the relative role of childhood negative events as a predisposing factor for e-mental health care. Previous studies found that childhood adverse experiences were associated in adulthood with higher care use, for somatic care [54], or for mental health care [55]. Our results, indicating a weaker utilization of e-mental health care, could signify less active treatment seeking among adults with a history of childhood adverse experiences and is associated with an increased risk of multiple health risk behaviors [56,57].

### Association With Traditional Services

As reported in previous studies, e-mental health care does not hinder traditional care [58]. We confirm that young people are “using online help seeking in combination with other services, rather than substituting online services for other resources” [24].

To the contrary, e-mental health care appears to be a step in the help-seeking process toward conventional mental health care. Several reasons can be proposed. First, e-mental health care could enhance mental health literacy [59]. Second, it could increase health empowerment and contribute to a more active attitude, which is necessary in psychotherapy [60]. The Spanish study among nursing students identified a profile of the few young adults (approximately 14%) who reviewed mental health information on the Internet instead of going to a doctor: mainly female, aged between 18 and 24 years, not living with their family, and living in the countryside [25]. In this Spanish student sample, the use of at least 1 online tool for mental health care concerned 97.7% of people having seen at least 1 health professional in the past year and 11.5% of those who has seen 1 psychologist or psychiatrist or counselor in the past year [25].

### Limitations

We acknowledge some limitations. The first is selective nonresponse in our community sample, which resulted in a higher proportion of young adults in high occupational grade jobs and with higher education than among young adults in France; nonetheless, unemployment rates in the study were comparable to the general population [61] and our main results were unchanged even when analyses were weighted to correct for factors associated with study participation. Second, we were not able to measure all Andersen’s model factors because we did not have information about important Internet-specific enabling factors (access, familiarity, and high-speed access) [6,50]. We hypothesize that they are unlikely to show high levels of variation and we were able to consider the model’s main factors [29]. Third, we lack information about the types of online services young people consulted and whether they were satisfied with the e-mental health care.

### Conclusion

In France, e-mental health care is a method of help-seeking behavior for young adults, even for those in the general population. Mental health professionals and policy makers must take note of its role. To begin, physicians have to figure out how best to use this fact to foster therapeutic alliance. They are in the best position to weigh information from the Internet and to advise patients in their particular situations [1].

## Abbreviations

ADHD: attention deficit and hyperactivity disorder
ASRS: ADHD Self-Report Scale
ESEMeD: European Study of the Epidemiology of Mental Disorders
GAZEL cohort: Gaz et Electricité cohort
GP: general practitioner
IReSP: Institut de la Recherche en Sante Publique
MINI: Mini-International Neuropsychiatric Interview
TEMPO cohort: Trajectoires Épidémiologiques en Population cohort
